# Spleen-Preserving Repair of Acute Gastric Volvulus With Wandering Spleen: Anterior Gastropexy and Splenopexy in a 20-Year-Old Man

**DOI:** 10.7759/cureus.115898

**Published:** 2026-09-07

**Authors:** Zachary J Tucker, Lucille Woodley

**Affiliations:** 1 General Surgery, Alabama College of Osteopathic Medicine, Dothan, USA; 2 General Surgery, AdventHealth Orlando, Orlando, USA

**Keywords:** anterior gastropexy, chromosome 22, gastric volvulus, minimally invasive laparoscopy, robot-assisted, robotic surgery, splenopexy, wandering spleen

## Abstract

Gastric volvulus is an uncommon condition in which the stomach twists on itself, and its association with wandering spleen is attributed to a shared defect of deficient peritoneal fixation. Both conditions are described predominantly in children, and reports of the combination in adults remain rare. When it does occur, the goal of management is often definitive fixation of the affected organs, yet there is limited guidance on the optimal approach in adults, particularly regarding splenic preservation. We present a rare case of gastric volvulus with wandering spleen in a 20-year-old man, managed with robot-assisted anterior gastropexy and splenopexy.

A 20-year-old man with autism and a reported chromosome 22 abnormality, described as a partial deletion with partial trisomy 22, presented with abdominal pain, distension, nausea, and nonbloody emesis. Family history was notable for a brother who died at age 13 from bowel ischemia secondary to volvulus. Computed tomography (CT) revealed extreme gastric distension with a wandering spleen located in the right upper quadrant. An upper gastrointestinal contrast study confirmed gastric volvulus at the pyloric channel. After nasogastric decompression with 3 L of output, the patient was taken to the operating room on hospital day two. Endoscopic reduction was unsuccessful, and robot-assisted reduction, anterior gastropexy, and splenopexy were performed. The spleen was viable and nonischemic at exploration, and fixation was chosen to preserve splenic function. The patient had an uncomplicated postoperative course and was discharged on postoperative day two.

Gastric volvulus with wandering spleen can present in young adults and should be considered when cross-sectional imaging demonstrates acute gastric obstruction with an ectopic spleen. Robot-assisted gastropexy and splenopexy addressed the underlying fixation defect while preserving splenic function. In this patient, communication difficulties related to autism complicated symptom assessment. Decisions to pursue advanced imaging should be guided by the clinical presentation and the degree of suspicion for volvulus rather than by any single patient characteristic.

## Introduction

Gastric volvulus refers to pathologic twisting of the stomach on itself, an uncommon condition that becomes rapidly lethal when rotation is severe enough to occlude the lumen and interrupt perfusion, resulting in ischemia and necrosis [1]. Gastric volvulus may be divided into primary and secondary forms, by axis of rotation into organoaxial, mesenteroaxial, and combined types, and by tempo into acute and chronic presentations [1]. Without prompt operative treatment, acute complete rotation carries a reported death rate of roughly one-third to one-half of cases [2]. This condition predominantly affects the extremes of age, with cases concentrated in children and older adults [3]. Presentation in a young adult is therefore atypical and may contribute to delayed recognition, particularly given the nonspecific clinical features that overlap with more common conditions such as gastritis and pancreatitis [1]. Cross-sectional imaging remains the primary diagnostic modality for gastric volvulus. In one comparative series with 10 participants with surgically confirmed gastric volvulus, the combination of an antropyloric transition point and an antrum positioned at or above the level of the fundus demonstrated 100% identification of volvulus cases while excluding all control cases [4]. 

Wandering spleen is characterized by the loss of the spleen’s normal peritoneal attachments, allowing it to move freely within the abdominal or pelvic cavity. Wandering spleen occurs across all ages. Roughly one third of reported cases are in children under 10 years, in whom the sex distribution is approximately equal, while among adults it is diagnosed most often between 20 and 40 years of age and far more frequently in women [5]. Wandering spleen and gastric volvulus arise from a shared defect of abnormal or absent intraperitoneal visceral attachment, confirmed directly through operative exploration, and the two conditions can aggravate one another because an untethered spleen destabilizes the poorly fixed stomach, worsening the resulting obstruction [5].

A spleen that is untethered by deficient ligaments leaves the stomach equally mobile, which has led to the recommendation of prophylactic gastropexy to reduce the risk of gastric volvulus [6]. In the largest reported surgical series of this association, of eight children with gastric volvulus requiring operative management, all patients had congenital splenic anomalies, including six with a wandering spleen. Combining minimally invasive gastropexy and splenopexy was effective, with only one recurrence attributed to suture failure [7]. Despite the shared mechanism, this combination is seldom reported, and the published experience is overwhelmingly pediatric. An early reported adult case, managed laparoscopically, was described in 2006 in a 67-year-old man [8]. 

No established genetic association currently links chromosome 22 abnormalities to gastric volvulus. The available chromosomal literature addresses intestinal rather than gastric rotation, with chromosome 22 alterations reported in association with malrotation and aganglionosis [9]. These cases occur as part of broader congenital syndromes and are accompanied by other congenital anomalies, and the few reports of gastric volvulus occurring alongside chromosomal disorders suggest coexistence rather than a direct causal relationship [10,11]. A more likely explanation in this case may be anatomical rather than genetic, with laxity of the peritoneal and ligamentous supports allowing excessive mobility of both the stomach and spleen [6].

We report a 20-year-old man with autism and a reported chromosome 22 abnormality, described by the family as a partial deletion with partial trisomy 22 but not confirmed by an available cytogenetic report, who presented with abdominal pain, nausea, and emesis and was found to have gastric volvulus with a wandering spleen, managed operatively with gastropexy and splenopexy after failed endoscopic reduction. To our knowledge, this association has not previously been reported in an adult with a chromosome 22 abnormality.

## Case presentation

The patient was a 20-year-old man with a past medical history of autism, a chromosome 22 abnormality reported by his mother as a partial deletion with partial trisomy 22 (the original cytogenetic report was not available for review), short stature, inguinal hernia as an infant, urethral reconstruction, and dental surgery, who presented with abdominal pain, distension, nausea, and nonbloody emesis. The patient’s mother stated that the patient complained of abdominal pain that began the evening prior to his presentation, as well as headaches for which ibuprofen did not provide relief. His abdominal pain persisted and coincided with the development of nausea and emesis. After repeated episodes of emesis, the patient was brought to the hospital by his mother. The patient’s mother denied previous episodes of similar symptoms but stated that he has had frequent belching and flatulence. The patient had not seen a gastroenterologist in the last few years and denied any prior esophagogastroduodenoscopy (EGD) or colonoscopy. At the time of presentation, he was not taking any medications and followed a regular diet. His last bowel movement was four days prior to presentation, which is his normal frequency, and he continued to pass flatus. Family history was notable for a brother who died at 13 years of age from sepsis secondary to bowel ischemia caused by volvulus. 

On evaluation in the emergency room, his WBC was 14,000 per microliter (reference range: 4,500 to 11,000 cells per microliter), and his vital signs were within normal limits. Abdominal exam revealed a soft, compressible abdomen with mild distension and tympany, tenderness in the right upper quadrant, and no peritoneal signs. Computed tomography (CT) showed marked gastric distension with an air-fluid level and a massively dilated stomach measuring 27 cm in length, with the pylorus not well visualized, as demonstrated in Figures 1-3. The differential included gastric outlet obstruction versus gastric volvulus. A nasogastric (NG) tube was placed with approximately 3 L of output. An upper gastrointestinal (GI) water-soluble contrast study demonstrated contrast in the proximal stomach with an obstructing transition at the pyloric channel consistent with gastric volvulus, as seen in Figure 4. The right upper quadrant position of the wandering spleen may have contributed through external compression, although this mechanism was not directly demonstrated through imaging.

**Figure 1:**
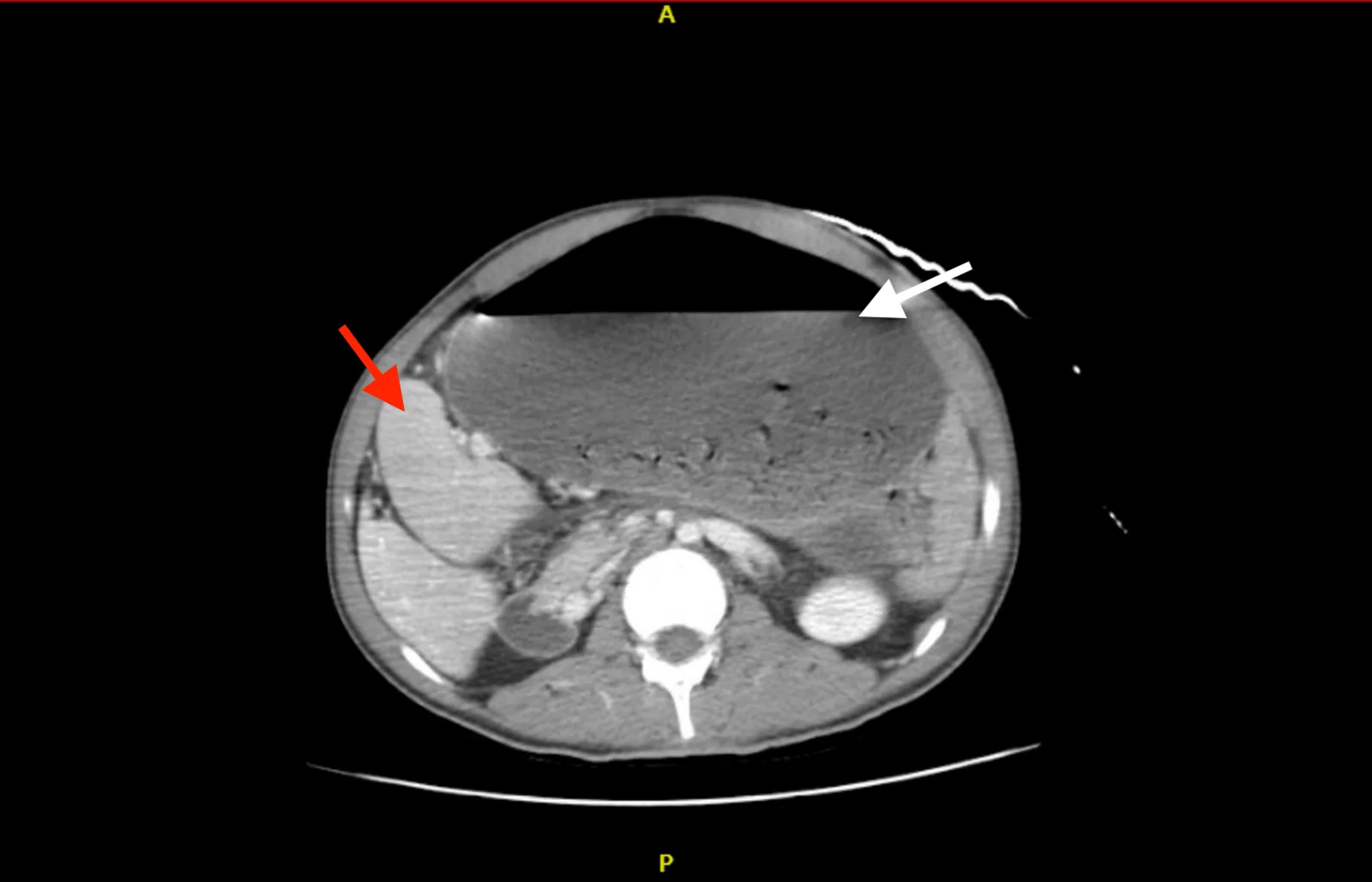
Axial computed tomography of the abdomen showing gastric volvulus (white arrow) and wandering spleen (red arrow).

**Figure 2:**
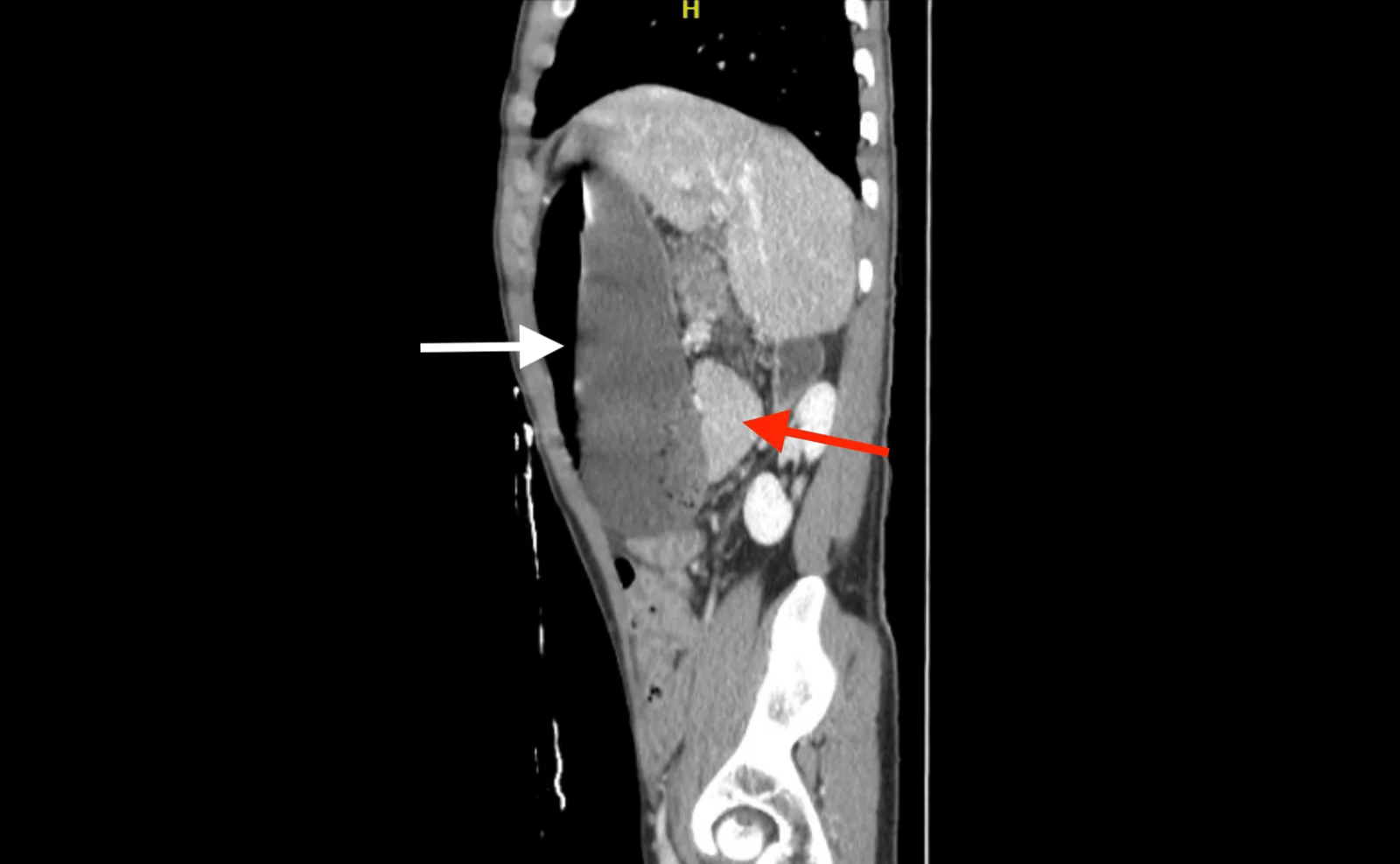
Sagittal computed tomography of the abdomen showing gastric volvulus (white arrow) and wandering spleen (red arrow).

**Figure 3:**
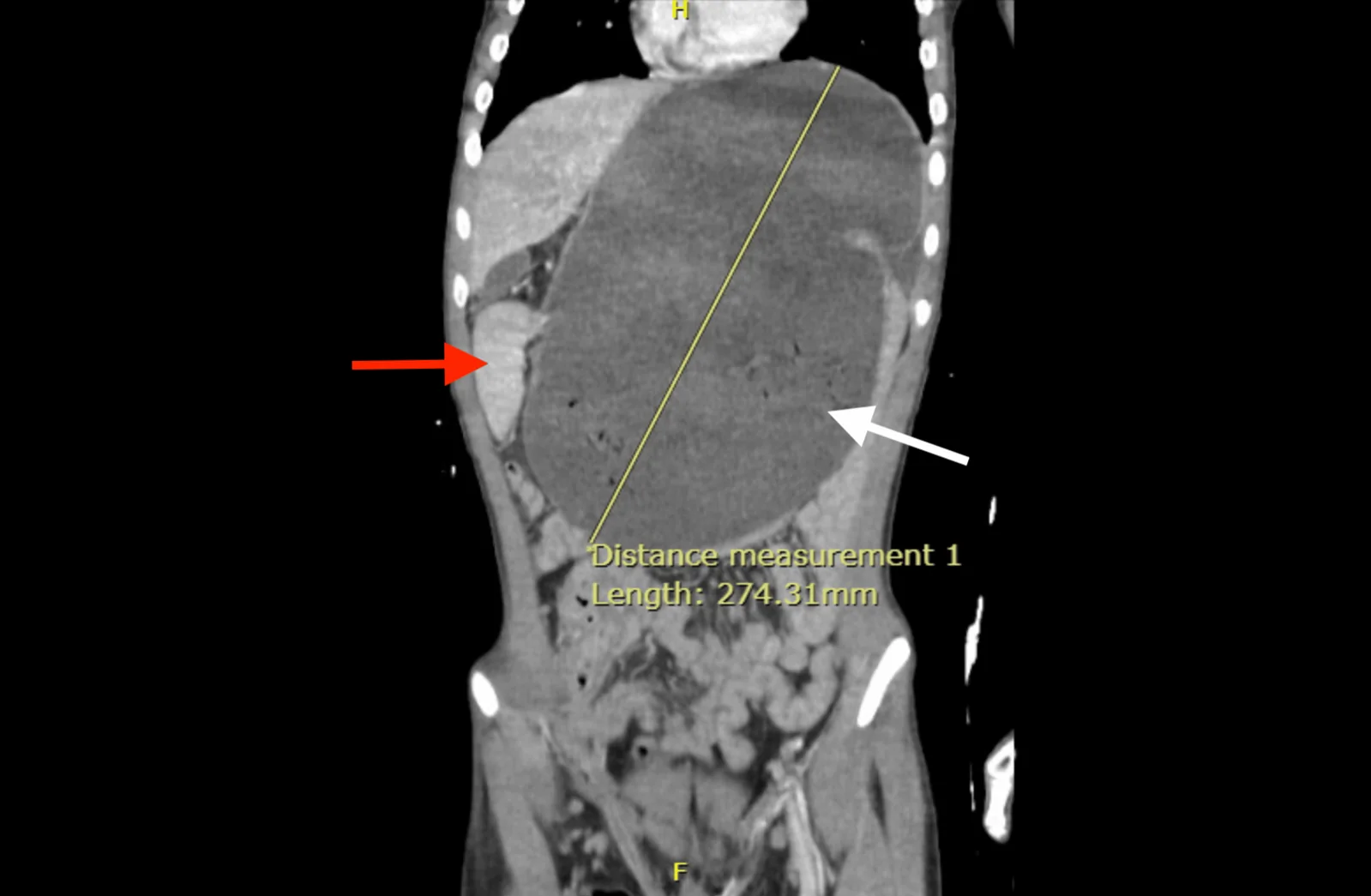
Coronal computed tomography of the abdomen showing gastric volvulus (white arrow) and wandering spleen (red arrow).

**Figure 4:**
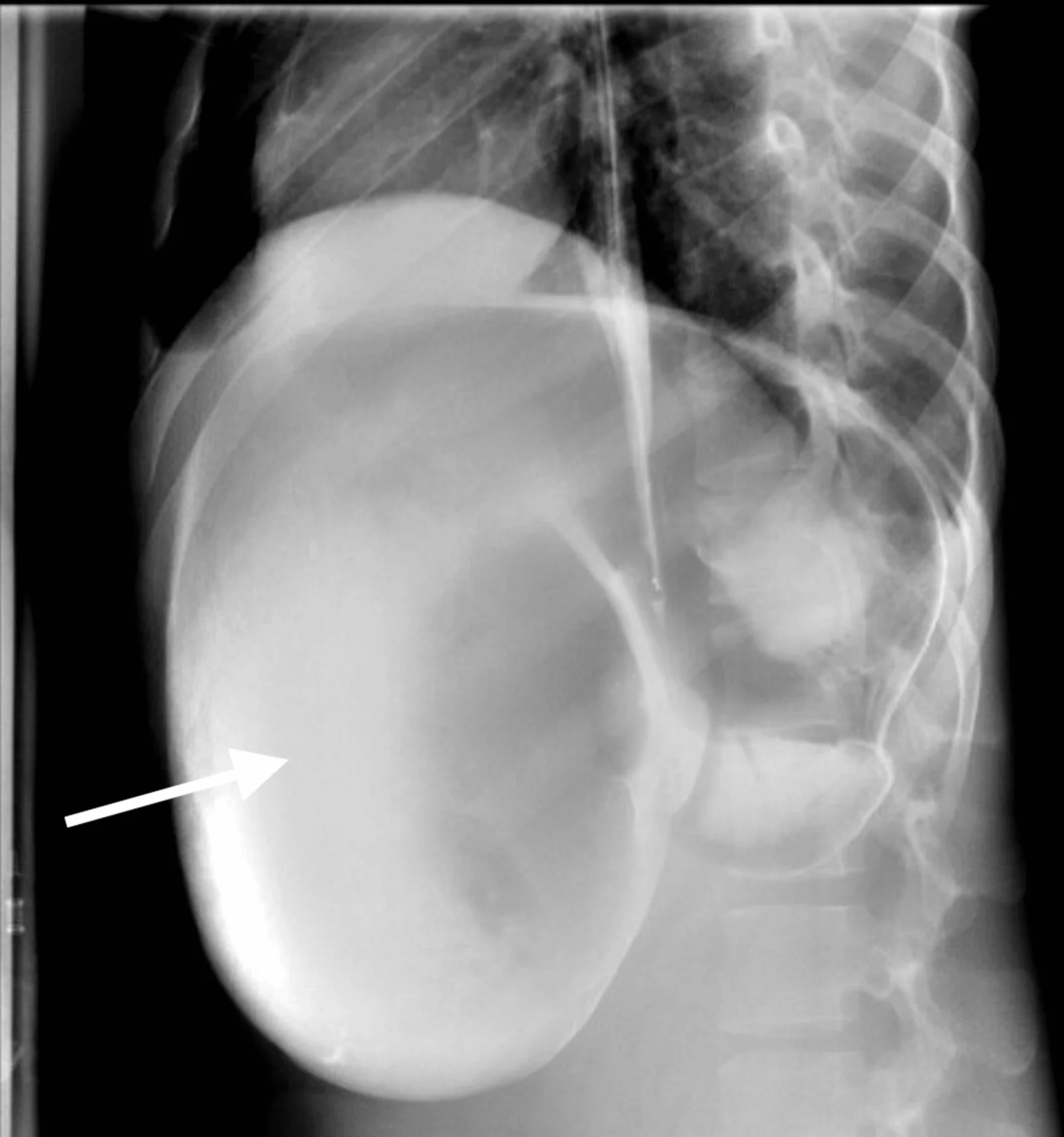
Upper GI series demonstrating gastric volvulus (white arrow).

Given the absence of peritonitis, only mild leukocytosis, and no signs of ischemia or perforation, and because the stomach had been successfully decompressed, the patient was managed non-emergently and taken to the operating room on hospital day two. The procedure started with an EGD, which confirmed the diagnosis of gastric volvulus. Attempted reduction with insufflation and gentle advancement of the scope failed to detorse the stomach, and the decision was made to enter the abdomen with the da Vinci Xi surgical system (Intuitive Surgical, Sunnyvale, CA, USA), beginning with a 5 mm Optiview port. After insufflation to 15 mmHg, the notably enlarged stomach was quickly identified, which appeared to be twisted. The specific direction and axis of rotation were not formally characterized at the time of operation. At this time, it was confirmed that the spleen had returned to its correct position in the left upper quadrant. Three additional 8 mm trocars were placed, and the initial 5 mm port was upsized to an 8 mm port. Using gentle traction, the gastric volvulus was reduced by rotating the stomach on its axis, and this was subsequently confirmed with an EGD, demonstrating that the pylorus was patent and the reduction was successful.

Following gastric detorsion, attention was then turned to the spleen. The spleen was found in its normal left upper quadrant position but had no lateral peritoneal attachments and demonstrated free mobility on gentle manipulation. This absence of ligamentous fixation with retained mobility confirmed the diagnosis of wandering spleen and provided the rationale for splenopexy. As the spleen appeared to be normal in color without evidence of infarction or torsion, a splenopexy was performed. The splenogastric ligament was mobilized and used to create the pexy, sutured to the lateral abdominal wall so that it lay snug over the spleen in situ, securing it against the lateral wall and eliminating movement. Gentle traction on the spleen confirmed that it was fixed in position.

The gastropexy was then conducted. A portion of the fundus was sutured to the peritoneum overlying the diaphragm, and the anterior greater curvature was sutured to the anterior abdominal wall. Visual inspection of the pylorus and antrum revealed patency and correct positioning. The abdomen was desufflated, and the incisions were injected with local anesthesia. The skin was closed with 4-0 Monocryl (Ethicon, Raritan, NJ, USA) sutures, and sterile dressings were placed. The patient tolerated the procedure well and was taken to the post-anesthesia recovery room in satisfactory condition. 

On postoperative day one, the patient and his mother reported that he was feeling well. He pulled out his NG tube overnight, but denied any nausea or vomiting, so the decision was made to discontinue the NG tube. The patient endorsed flatus and a bowel movement. The remainder of his hospital course was uncomplicated, and he was discharged on postoperative day two.

The patient did not attend his scheduled two-week follow-up visit. No in-person follow-up examination was obtained, and longer-term follow-up is not available.

## Discussion

This case illustrates gastric volvulus with wandering spleen in a young adult, a combination that has been described almost exclusively in children and only rarely in adults, with an early reported adult case managed laparoscopically in 2006 [8]. The likely underlying abnormality is deficient peritoneal fixation rather than an acquired process. Whether the patient's chromosome 22 abnormality contributed to this developmental fixation defect cannot be determined from the available data, but such a contribution remains possible. Consequently, the loss of normal splenic ligamentous attachments is associated with increased gastric mobility, providing the rationale for addressing both organs during surgical intervention [6]. In the largest surgical series examining this association, all children who underwent operative treatment for gastric volvulus were found to have a congenital splenic anomaly. Combined minimally invasive gastropexy and splenopexy have been reported as effective, with only a single recurrence reported, which was attributed to suture failure [7]. Contemporary expert practice holds that conversion to an open approach or primary laparotomy remains appropriate when gastric necrosis or perforation is suspected, as these complications define a high-risk subgroup associated with mortality of approximately 30 percent [12]. 

Cross-sectional imaging established the diagnosis in the present case, consistent with findings from a retrospective emergency series in which computed tomography was diagnostic in all 26 patients who underwent imaging, whereas upper endoscopy identified the condition in only 9 of 20 cases [12]. When endoscopic reduction is unsuccessful, definitive surgical management rather than repeated endoscopic attempts is appropriate; this has been described in chronic gastric volvulus with symptom resolution following laparoscopic anterior gastropexy [13]. Endoscopy nevertheless retains an important role, both for facilitating gastric decompression and, in other reports, for evaluating mucosal viability, although in the present case it served to confirm the volvulus and attempt reduction rather than to formally assess the mucosa [6].

The spleen was viable and nonischemic at exploration, so splenectomy, which has historically been the default operation for symptomatic wandering spleen, was not indicated. Splenopexy addressed the underlying problem of uncontrolled splenic mobility while preserving immune function given his expected long-term risk of postsplenectomy infection. Fixation also complements the gastropexy, because a mobile spleen in the right upper quadrant contributed to the abnormal gastric position in this case, and leaving it untethered would preserve one of the forces predisposing to recurrent rotation. Splenopexy combined with gastropexy has been effective in the reported pediatric experience, where the only recurrence followed suture failure rather than failure of the planned procedure itself [7]. 

Chromosome 22 abnormalities have a recognized gastrointestinal phenotype, best characterized in 22q11.2 deletion syndrome, in which chronic functional gastrointestinal symptoms were reported in 91% of 206 adults over their lifetime while congenital gastrointestinal malformations occurred in 3.5% [14]. Whether the present patient’s rearrangement involves the 22q11.2 region could not be determined, as the original cytogenetic report was unavailable; nevertheless, structural foregut anomalies are not an established feature of chromosome 22 disorders. The chromosomal reports that do involve rotation and fixation are confined to the intestine and occur alongside other anomalies [9]. Taken together, these findings do not support a causal chromosomal mechanism in the present case, and the operative findings of deficient peritoneal fixation of both the stomach and spleen offer the more likely explanation for both the gastric volvulus and wandering spleen.

A limitation of this report is that the original cytogenetic data, including breakpoints and method of analysis, were unavailable, preventing confirmation of whether the rearrangement involves regions with known gastrointestinal associations and limiting the ability to draw conclusions about a genetic contribution. A further limitation is the absence of clinical follow-up. The patient did not attend his scheduled two-week visit, and no longer-term follow-up was obtained, so the durability of the repair and freedom from recurrence cannot be assessed. Conclusions are therefore limited to the immediate operative and early postoperative period.

The family history is notable in this case. According to the patient's mother, his brother died at 13 years of age from sepsis secondary to bowel ischemia caused by volvulus, raising the possibility of a familial defect of visceral fixation, although the sibling's original medical and operative records were not available for independent review. Inadequate mesenteric attachment predisposes to volvulus wherever it occurs, and the sibling’s intestinal involvement and the present patient’s gastric involvement are compatible with a single underlying failure of peritoneal fixation that manifests at different levels of the gastrointestinal tract. Whether this familial pattern relates to the chromosome 22 rearrangement cannot be determined from the available information. The practical implication is that cross-sectional imaging should be obtained early when a patient with a chromosome 22 abnormality or a family history of volvulus presents with acute abdominal symptoms, since chronic functional complaints are common in patients with chromosome 22 disorders and a sudden change in clinical course should not be attributed to that baseline pattern. Communication challenges associated with autism may further complicate assessment, supporting early imaging when physical findings appear disproportionate to the reported symptoms.

## Conclusions

Gastric volvulus with wandering spleen is a rare but recognized association that should be considered in any patient presenting with acute gastric obstruction and an ectopic spleen, both of which are readily demonstrated on contrast-enhanced CT of the abdomen. This case demonstrates that the combination can present in a young adult and be successfully managed with robot-assisted gastropexy and splenopexy, with preservation of the viable spleen while addressing the underlying defect of peritoneal fixation. Long-term durability cannot be assessed given the absence of follow-up. The family history of fatal intestinal volvulus in a sibling raises the possibility of a shared developmental abnormality of visceral attachment and underscores the importance of maintaining a low threshold for obtaining advanced imaging in patients with a personal or family history of volvulus.

## References

[1] Chiarenza SF, Zolpi E, Costa L, Cocco P, Conighi ML, Bleve C (2022). Pediatric gastric volvulus: is laparoscopic Hill-Snow-modified gastropexy the effective long-term minimally invasive solution?. J Laparoendosc Adv Surg Tech A.

[2] Yang Z, Xie X, Wang S, Pei G, Zhan J (2024). An acute gastric volvulus in a child with congenital left diaphragmatic hernia: a case report. BMC Pediatr.

[3] Cribbs RK, Gow KW, Wulkan ML (2008). Gastric volvulus in infants and children. Pediatrics.

[4] Millet I, Orliac C, Alili C, Guillon F, Taourel P (2014). Computed tomography findings of acute gastric volvulus. Eur Radiol.

[5] Lin CH, Wu SF, Lin WC, Chen AC (2005). Wandering spleen with torsion and gastric volvulus. J Formos Med Assoc.

[6] Spector JM, Chappell J (2000). Gastric volvulus associated with wandering spleen in a child. J Pediatr Surg.

[7] Nakagawa Y, Uchida H, Amano H (2022). Patients with gastric volvulus recurrence have high incidence of wandering spleen requiring laparoscopic gastropexy and splenopexy. Pediatr Surg Int.

[8] Matsushima K, Kayo M, Hachiman H, Gushimiyagi M (2006). Laparoscopic repair of gastric volvulus associated with wandering spleen in an adult: report of a case. Surg Today.

[9] Hall T, Samuel M, Brain J (2009). Mosaic trisomy 22 associated with total colonic aganglionosis and malrotation. J Pediatr Surg.

[10] Martin V, Shaw-Smith C (2010). Review of genetic factors in intestinal malrotation. Pediatr Surg Int.

[11] Kotobi H, Auber F, Otta E, Meyer N, Audry G, Hélardot PG (2005). Acute mesenteroaxial gastric volvulus and congenital diaphragmatic hernia. Pediatr Surg Int.

[12] Light D, Links D, Griffin M (2016). The threatened stomach: management of the acute gastric volvulus. Surg Endosc.

[13] Lee HY, Park JH, Kim SG (2015). Chronic gastric volvulus with laparoscopic gastropexy after endoscopic reduction: a case report. J Gastric Cancer.

[14] Kotcher RE, Chait DB, Heckert JM (2022). Gastrointestinal features of 22q11.2 deletion syndrome include chronic motility problems from childhood to adulthood. J Pediatr Gastroenterol Nutr.

